# High SARS-CoV-2 secondary infection rates in households with children in Georgia, United States, Fall 2020—Winter 2021

**DOI:** 10.3389/fpubh.2024.1378701

**Published:** 2024-10-25

**Authors:** Kacy D. Nowak, Morgan A. Lane, Armand Mbanya, Jasmine R. Carter, Brianna A. Binion, Daniel O. Espinoza, Matthew H. Collins, Christopher D. Heaney, Nora Pisanic, Kate Kruczynski, Kristoffer Spicer, Magdielis Gregory Rivera, Felicia Glover, Tolulope Ojo-Akosile, Robert F. Breiman, Evan J. Anderson, Felipe Lobelo, Jessica K. Fairley

**Affiliations:** ^1^Hubert Department of Global Health, Rollins School of Public Health, Emory University, Atlanta, GA, United States; ^2^Division of Infectious Diseases, Department of Medicine, Emory University School of Medicine, Atlanta, GA, United States; ^3^The Southeast Permanente Medical Group, Department of Quality and Patient Safety, Kaiser Permanente of Georgia, Atlanta, GA, United States; ^4^Department of Epidemiology, Rollins School of Public Health, Emory University, Atlanta, GA, United States; ^5^Department of Environmental Health and Engineering, Johns Hopkins Bloomberg School of Public Health, Baltimore, MD, United States; ^6^Department of Epidemiology, Johns Hopkins Bloomberg School of Public Health, Baltimore, MD, United States; ^7^Department of International Health, Johns Hopkins Bloomberg School of Public Health, Baltimore, MD, United States; ^8^Center for Childhood Infections and Vaccines, Children’s Healthcare of Atlanta and Emory University School of Medicine, Atlanta, GA, United States; ^9^Division of Infectious Diseases, Department of Pediatrics, Emory University School of Medicine, Atlanta, GA, United States

**Keywords:** severe acute respiratory syndrome coronavirus 2, COVID-19, pediatric COVID-19, household and family, communicable disease transmission, SARS-CoV-2

## Abstract

**Background:**

A wide range of household secondary infection rates has been reported, and the role of children in population transmission dynamics for SARS-CoV-2 remains ill-defined. We sought to better understand household infection early in the pandemic.

**Methodology:**

A cross-sectional study of 17 households in the Atlanta metropolitan area with at least one child and one case of COVID-19 in the prior 1–4 months were recruited between December 2020 and April 2021. Self-collected saliva samples were tested on a multiplexed platform to detect IgG antibodies that bind to SARS-CoV-2 antigens. Secondary infection rates (SIR) were calculated and compared.

**Results:**

We report results on 17 families, including 66 individuals. We found an average SIR of 0.58; children and adults were similarly infected (62% children vs. 75% adults) (*p* = 0.2). Two out of 17 households had a pediatric index per our definition. Number of pediatric infections per household (*p* = 0.18), isolation (*p* = 0.34), and mask wearing (*p* = 0.80) did not differ significantly among households with an SIR above the mean vs. those with SIR below the mean. Households with higher SIR also had a higher number of symptomatic cases (*p* < 0.001).

**Discussion:**

We demonstrated high household SIRs at the early stages of the pandemic in late 2020 to early 2021 with similar impact on children and adults. The ease of collecting saliva and the detection of asymptomatic infections highlight the advantages of this strategy and potential for scale-up.

## Introduction

Elucidating the role children and adolescents play in the transmission of SARS-CoV-2 as well as the impact of household transmission on them has been a challenge due to under testing, school closures at the start of the pandemic, and a high estimated proportion of asymptomatic pediatric infections (16–50%) (1). However, as the pandemic continued, pediatric cases rose due to evolving viral genetics and relaxation of non-pharmacological interventions. The American Academy of Pediatrics (AAP) reported that as of January 2022, almost 8.5 million (17.4% of total cases) pediatric cases of SARS-CoV-2 infection were diagnosed in the United States (U.S) (2). Hospitalization and severe COVID-19 infection rates among children rose as the Delta variant surged, and increased further with the emergence of the Omicron variant. Although pediatric SARS-CoV-2 hospitalization rates have been lower compared to older age groups, pediatric hospitalization rates went up significantly with the Omicron wave due to the sheer number of infections (3).

SARS-CoV-2 household transmission studies have ranged in methodology and outcomes. At the beginning of the pandemic, studies showed relatively low secondary infection rates (6–51%), but these were predominantly in Asia where index cases were often isolated from the rest of the household (4). As Europe and North America began to study household transmission, SIR were found to be consistently higher than those initial estimates, with an SIR up to 70% found in minority households in North Carolina (5–13). At the same time, while the likelihood of transmission from pediatric case was debated and found to have mixed results, several studies concluded that children were not primary drivers of transmission and that schools were safe to reopen (14–16). However, SIR of pediatric vs. adult index cases was found to be similar in situations where the index case could be identified (10–12, 17). And while a more recent meta-analysis published in 2022 analyzing 95 articles (48 studies included in the meta-analysis) demonstrated a pooled household SIR from pediatric index cases lower than that of adult index cases [0.20 (95% CI 0.15–0.26) vs. 0.64 (95% CI 0.64–0.85)], this difference did not hold up with more recent SARS-CoV-2 viral variants (18).

However, the majority of household transmission studies involved either retrospective identification of symptomatic cases or time-intensive longitudinal studies during the acute symptomatic infection phase (5, 19, 20). Both approaches likely missed a substantial proportion of infections and therefore biased secondary infection rates (SIR) estimates. Use of serology can improve asymptomatic case detection with select studies demonstrating SIR in children ranging from 7.6 to 48% when serology was used (7, 9, 21).

In Georgia, USA, data on household transmission, especially in households with children, have been limited. Previous studies have provided some insight but often lacked comprehensive testing strategies, such as serological testing, to accurately determine secondary infection rates (SIR). For instance, a survey of households with pediatric index cases reported an average SIR of 45.7%, which is likely an underestimate due to the reliance on symptomatic testing alone (22). Give these gaps, our cross-sectional study aimed to investigate secondary infection rates SARS-CoV-2 infection during the early stages of the pandemic in households with children using a saliva-based IgG test and compare SIRs according to individual and household-level characteristics. We hypothesized that children in households would be infected with SARS-CoV-2 at similar rates as the adult members and that index cases would not be solely confined to adults. Our findings have the potential to inform targeted public health strategies and interventions, especially in managing and preventing the spread of COVID-19 within households. By understanding that children can be infected at similar rates as adults and recognizing that index cases are not confined to adults, we can better design measures to protect all household members and curb the spread of the virus more effectively. This study contributes valuable data to the ongoing efforts to understand and combat COVID-19, particularly in family and household settings where the risk of transmission is high.

## Methods

### Study setting and participants

For this cross-sectional study, households were recruited from December 2020 to April 2021 and were identified through adult individuals who tested positive for SARS-CoV-2 infection at outpatient screening clinics at Emory Healthcare and through study advertisements on social media and university listservs. Households were eligible to participate if at least one member was under the age of 18 years and if one or more individuals tested positive for SARS-CoV-2 within 4–16 weeks prior to the study visit (dates of infection were late September 2020 through February 2021). This study was performed in a 1-4-month window to limit the likelihood that a subsequent SARS-CoV-2 infection, in the interim. All household members needed to agree to participate for the household to be eligible. Reported information about the index case such as timing of SARS-CoV-2 testing was collected retrospectively. At the time of this study, repeated infection was very rare. However, we did exclude any household members who reported a previous known infection.

### Data collection and materials

Individuals then self-collected saliva samples using an Oracol™ device by rubbing a sponge in the gingival space on both sides of the participant’s mouth for 1 minute. Saliva samples were processed and then tested on a Luminex platform for IgG antibodies that bind to the SARS-CoV-2 nucleocapsid (N), receptor binding domain (RBD), and spike (S) proteins as previously described (23). A sample was defined as SARS-CoV-2 IgG positive if reactive to the GenScript N antigen and either the RBD or S Spike proteins as previously determined during validation of the assay (23). Total IgG was measured in samples testing negative for SARS-CoV-2 IgG to exclude false negatives due to insufficient sample quality. Samples without meeting a validated threshold of total IgG were classified as indeterminate. Since this study was conducted earlier in the pandemic, repeated infections were less common, and most individuals had not been infected more than once yet due to remote schooling, social distancing, and masking efforts. Therefore, detected antibodies were unlikely due to previous infections occurring before the study time period, and were thus considered as an infection occurring during the household transmission period. Individual and household surveys were completed electronically in RedCap by individuals after the study visit (24) and included individual demographics, symptoms, pre-existing medical conditions, exposures, and a household survey describing infection timelines and isolation procedures in the household.

### Study variables

A case was defined as a household member with a reported positive molecular test for SARS-CoV-2 result, a reported positive SARS-CoV-2 antigen test, or a positive SARS-CoV-2 salivary antibody test. A household index case was defined as the case who received the first confirmed SARS-CoV-2 test result or had the earliest symptom onset date among the household cluster. A household contact was defined as anyone living in the same residence as the index case at the time of infection. The outcome variable assessed was the secondary infection rate (SIR) which was defined as the proportion of secondary cases in a household infected from the index case over the total number of household contacts. The SIR was then dichotomized in order to compare households with an SIR below the study sample mean and households above the mean in order to identify factors association with a higher SIR.

### Statistical analysis

Statistical analysis was performed using a t-test or Fisher’s Exact test as appropriate to explore differences between household SIR and household characteristics during the time of infection. Independent variables included whether the index case was an adult (≥18 years old) or child, number of individuals in the household, whether the index case was symptomatic, and whether bedrooms and/or bathrooms were shared in the household at the time of infection. While this was an exploratory pilot study, to assess the adequacy of the sample size, we estimated that SIR in households with adult index cases would be 4 times more likely to have a high SIR compared to households with pediatric index cases. If adult index cases were twice as common as pediatric, and assuming a power of 0.80, and 95% significance level, a sample size of 24 households would be ideal. All statistical analyses were performed using Rstudio 1.3.1073.

## Results

Seventeen households were enrolled with a total of 66 individuals, 34 (52%) of which were under 18 years old. The median age was 15.5 (IQR = 32.3), with 30 (46%) males and 36 (55%) females. Fifty-five of the 66 individuals (83%) reported testing at the time of acute infection, of which 35 (64%) were positive for SARS-CoV-2 molecular or antigen testing.

From the 66 saliva samples tested, 42 (64%) were positive for SARS-CoV-2 IgG antibody, 20 (30%) negative, and 4 (6%) were indeterminate due to low total IgG in the saliva sample. These 4 indeterminates were children who were either negative for SARS-CoV-2 PCR testing or did not get tested, therefore, we excluded them from the analysis given the uncertainty of their infection status. Six individuals with a negative SARS-CoV-2 PCR test were positive for SARS-CoV-2 IgG as defined in the methods. Among cases who reported a positive SARS-CoV-2 molecular or antigen test, four individuals (11.4%) received negative antibody test results. Furthermore, we were able to use antibody test results to assign infection status to nine individuals who did not receive SARS-CoV-2 testing at the time of household infection (five had positive antibodies and four had negative antibodies). Including self-reported SARS-CoV-2 test results (antigen or PCR) and salivary antibody test results, 46 individuals (70%) were defined as having had SARS-CoV-2 infection. The average household positivity of SARS-CoV-2 infections for the 17 families was 0.71 (SD = 0.3), meaning on average, 71% of household members were infected during the household episode. The average SIR was 0.58 (SD = 0.4) and the median SIR was 0.67 (range 0–1.0). Twenty-one children were infected (62% of children) versus 25 adults (78% of adults) (*p* = 0.19). All positive SARS-CoV2 cases known at the time of infection had either a COVID test or onset symptoms within 8 days of the household index case.

Among those who were infected, the average symptomatic case frequency in each household was 0.66 (SD = 0.4). Six children were asymptomatic (29% of infected children) and four adults were asymptomatic (16% of infected adults). The median age of the 49 household contacts was 10 (IQR = 29) years and for the 17 index cases was 40 (IQR = 13.0) years with nine being female. Fifteen (88%) index cases were symptomatic and four (24%) reported a comorbid chronic medical condition.

Children under 18 years made up 52% of the study sample but only two (12%) of the index cases. The ages of the child index cases were three and 15. The household SIR for the child index cases were 1.0 and 0.43 (average = 0.71). Both child index cases attended either in-person daycare or school (one of which had a known school exposure). There were more symptomatic cases in the high SIR group vs. low (Table 1), although not controlled for household size (on average 3 vs. 1, *p* < 0.001). There was no significant difference in isolation and masking behaviors between the low and high SIR groups (Table 1). No participants were fully vaccinated at the time of the household infection, although three adults were within 1 week after the first dose of an mRNA vaccine when the household infection occurred.

**Table 1:** Individual and household demographics stratified by average secondary infection rate (SIR).

Demographics	Above average SIR	Below average SIR	Overall	*p* value
Household-level characteristics				
	**(*n* = 10)**	**(*n* = 7)**	**(*n* = 17)**	
**Household size**				0.81
Mean (SD)	3.80 (0.63)	4.00 (2.0)	3.88 (1.32)	
Median (IQR)	4.00 (0.75)	3.00 (1.5)	4.00 (0.75)	
**Number of pediatric infections n (%)** ^*^	18 (53)	3 (25)	21 (46)	0.18
**Symptomatic cases per household**				**<0.001**
Mean (SD)	3.0 (0.7)	1.1 (1.07)	2.2 (1.3)	
Median (IQR)	3.0 (0.0)	1.0 (1.0)	3.0 (2.0)	
**Index case isolation, n (%)**	6 (60)	6 (86)	12 (71)	0.34
**Bedroom/bathroom sharing with index case, n (%)**				0.54
Only a bedroom	0 (0)	0 (0)	0 (0)	
Only a bathroom	1 (10)	1 (14)	2 (12)	
Both	6 (60.0)	2 (28.6)	8 (47.1)	
Neither	3 (20.0)	4 (57.1)	7 (41.2)	
**Mask use in household, *n* (%)** ^**^				0.80
All of the time	3 (30.0)	3 (42.9)	6 (35.3)	
Most of the time	2 (20.0)	1 (14.3)	3 (17.6)	
Occasionally	1 (10.0)	0 (0.0)	1 (5.9)	
Rarely	1 (10.0)	1 (14.3)	2 (11.8)	
Never	3 (30.0)	2 (28.6)	5 (29.4)	
Index case characteristics				
	**(*N* = 10)**	**(*N* = 7)**	**(*N* = 17)**	
**Age (years)**				0.83
Mean (SD)	38.6 (14.0)	37.1 (13.0)	36.7 (15.1)	
Median (IQR)	41.5 (9.5)	40 (14.5)	40 (13.0)	
**Child index case, n (%)**	1 (10.0)	1 (24.3)	2 (11.8)	0.82
**Sex, n (%)**				0.33
Female	4 (40.0)	5 (71.4)	9 (52.9)	
**Race/ethnicity, n (%)**				0.73
White	5 (50.0)	5 (71.4)	9 (52.9)	
Hispanic/Latino	1 (10.0)	0 (0.0)	1 (5.9)	
Black/African American	3 (30.0)	1 (14.3)	4 (23.5)	
Asian	0 (0.0)	1 (14.3)	1 (5.9)	
Mixed Race	1 (10.0)	0 (0.0)	1 (5.9)	
**Co-morbidities present, *n* (%)**	2 (20.0)	2 (28.6)	4 (23.5)	0.72
**Symptomatic, *n* (%)**	10 (100.0)	5 (71.4)	15 (88.2)	0.15
Household secondary cases characteristics				
	**(*N* = 24)**	**(*N* = 5)**	**(*N* = 29)**	
**Age (years)**				0.23
Mean (SD)	17.4 (15.8)	27.4 (19.6)	19.1 (16.6)	
Median (IQR)	9 (28.25)	33 (26.0)	11 (29.0)	
**From a household with child index case, *n* (%)**	4 (16.7)	3 (60.0)	7 (24.1)	0.07
Sex, *n* (%)				
Female	15 (62.5)	1 (20.0)	16 (55.2)	0.14
**Race/Ethnicity, *n* (%)**				0.31
White	13 (54.2)	4 (80.0)	17 (58.6)	
Hispanic/Latino	3 (12.5)	0 (0.0)	3 (10.3)	
Black/African American	5 (20.8)	0 (0.0)	5 (17.2)	
Asian	0 (0.0)	1 (20.0)	1 (3.4)	
Mixed Race	3 (12.5)	0 (0.0)	3 (10.3)	
**Co-Morbidities, *n* (%)**	7 (29.2)	1 (20.0)	8 (27.6)	0.76
**Symptomatic, *n* (%)**	18 (75.0)	3 (60.0)	21 (72.4)	0.60

‘n’ represents household-level population and ‘N’ represents individual-level population. The average secondary infection rate (SIR) was 0.579; *p* values were estimated with fisher’s exact test for categorical variables and *t*-test for continuous variables.

* Percentages used total number of COVID-19 cases in each column rather than total individuals in each column.

** *P-value for mask use was determined by mid-P exact test comparing “all of the time” and “most of the time” vs none, rare, or occasional use.Bolded values signify a significant p-value, < 0.05.*

## Discussion

We found an overall high secondary household infection rate of 58%, with 62% of children and 78% of adults infected with SARS-CoV-2. While small in scope, observing children who are both becoming infected and transmitting SARS-CoV-2 (in the case of likely index cases) to multiple household members is notable and aligns with higher estimates of SIR in the literature (9, 14). As observed with other respiratory viruses, our data also highlight the potential risk for children initiating transmission clusters among their close relatives, including those that may be a higher risk for adverse COVID-19 outcomes due to age and other medical comorbidities. Since repeated infections were not common at the time of the study, we assume that most of the “asymptomatic antibody positive” infections happened during the household infection. Not only did early studies have lower SIR (6–51%) than what we found, they also concluded that pediatric index cases were associated with lower household transmission (9, 14, 16, 25, 26). These studies that identified lower fewer pediatric than adult index cases were likely impacted by school closures and limited by methodologic factors such as under testing of asymptomatic cases. Additionally, many studies did not enroll the entire household which may have resulted in underestimation of SIR (5, 22, 27). While we had many more households with adult index cases, the SIR did not differ by adult vs. pediatric index case nor were children less likely to be infected compared to adults.

Our SIR was more in line with European and North American studies early in the pandemic where SIR were found to be to be consistently higher than those initial estimates, like a study that found an SIR of 70% found in in North Carolina (5–13).

Interestingly, household-level isolation measures did not differ between high or low SIR, but these data were limited by an overall small number of households. This was not consistent with other studies, many of which showed statistically significant associations between NPI usage (isolation, mask usage) and lower secondary transmission in households (9, 28–30). It is possible that in some of our cases, household transmission had already occurred before infection status was known, and therefore, before household mitigation activities could have prevented spread. A larger sample size may be needed to differentiate the potential effect of mitigation on the likelihood of household transmission. As no participants were fully vaccinated during the time of the household infection, vaccination did not impact household transmission. Furthermore, since the IgG positivity was dependent on the N antigen (not a target of the available vaccines in the U.S) it did not matter if individuals were vaccinated between the household infection and the study visit. While this analysis was pre-Delta and pre-Omicron, we would expect even higher secondary infection rates with Delta and Omicron than we found in this study, albeit possibly mitigated in part due to more widespread vaccination.

Our study had 10 asymptomatic cases, two of which were definite index cases. Both index cases were the sole case in their household which allowed for a confident assignment of these index cases. Other studies have also found asymptomatic cases when antibody tests are used (31). Households in our study with higher SIR also had more symptomatic cases (3 vs. 1, *p* = 0.001), This could suggest the presence of more transmissible virus in the household even if the index case being symptomatic did not differ between high and low SIR (*p* = 0.15). Prior studies have been mixed to whether symptomatic index cases were associated with higher SIR (26, 32). A larger sample size may have better defined both of these factors. Other index case characteristics aside from age and symptom status, like race and co-morbidity also did not differ among households with high and low SIR.

Lastly, the findings of this study demonstrate the challenge of precisely defining the role children play in the transmission of SARS-CoV-2, because if children are asymptomatic and either do not get tested or test negative but are truly a case, many pediatric cases go unreported and onward transmission unrecognized. We recognize that household transmission dynamics became more complicated as the pandemic progressed beyond 2021 with repeat infections, vaccinations, and more transmissible variants. That being said, this project benefited from the lack of these complicating factors and is informative in situations and locales with low vaccination rates, low prior infections, and potential future variants with high degrees of immune evasion.

Overall, this study demonstrated a high household SIRs in late 2020 / early 2021 in Atlanta, GA, where proportions of children and adults acquiring infection in the household were similar. In addition, pediatric index cases resulted in multiple subsequent household infections. Recruiting entire households and collecting saliva swabs for antibody tests on all members (infants and up) was convenient and feasible as it did not require invasive phlebotomy or a skilled research nurse. The ease of sample collection was a major outcome of the study and demonstrates the scalability and acceptability of this epidemiologic study design. The multiplex assay also has the advantage of differentiating between natural and vaccinated individuals, which is important in studying transmission in the setting of vaccination.

We also overcame methodologic limitations of previous studies, which were unable to account for asymptomatic infections given challenges of drawing blood in children, by employing a multiplexed saliva-based antibody assay that highly correlates with serum antibodies (15). In addition, the sensitivity and specificity of the assay (98 and 99%, respectively, with the N antigens used in this study) as well as validation tests showing minimal concern about cross-reactivity with common human coronaviruses, also support the use of the assay (23). This scalable, minimally invasive tool can complement household transmission studies and provide a more acceptable alternative to venipuncture in young children, with the ultimate goal to better inform public health policies especially those involving children, school, and vulnerable populations. While some of the benefits of an antibody tool are lost with repeat infections in the same individual, refinement of the antigen–antibody combinations, like the use of IgM or antigens that differ across variants, could maintain utility of this multiplexed approach. Determining the frequency of asymptomatic infections, especially as variants emerge, is important as there are still populations, such as older adults and immunocompromised, who can have poor outcomes from SARS-CoV-2 infection.

Our study had several limitations. Primarily, the results are descriptive, the study design is cross-sectional, and we acknowledge the lack of inferential statistical analyses to identify the relationships between dependent and independent variables. This limitation is partly due to the constraints of our sample size, which restricts the extent of statistical analysis we can perform. Despite these limitations, we have conducted univariate comparisons to the best of our ability given the sample. This study focused on describing the early days of the pandemic in households with COVID-19 in Georgia, USA, during Fall 2020 to Winter 2021. Ethical approvals restricted us from collecting data from screened individuals who did not agree to participate, hindering our understanding of potential selection biases and affecting the generalizability of our findings. The different recruitment methods may have introduced biases that are difficult to quantify and account for in our analysis. Lastly, our sample size being shy of the calculated ideal of 24 households, misclassification of the index case, and the possibility that a family member had been previously infected at an earlier point in the pandemic may have impacted our results. An index case could have also been asymptomatic and not discernible in our study since antibody tests cannot determine temporality.

The SARS-CoV-2 pandemic is not over and other viral pandemic are predicted in the near future. Having demonstrated the feasibility of studying household SARS-CoV-2 infections with this non-invasive assay, it can also be used as a model for studying the next highly contagious virus like SARS-CoV-2. To improve the robustness and accuracy of our results, future studies should aim to maximize the sample size and incorporate more comprehensive inferential statistical methods. We also recommend longitudinal studies for documenting causality.

## Data Availability

The raw data supporting the conclusions of this article will be made available by the authors, without undue reservation.
